# Effects of the Deslagging Process on some Physicochemical Parameters of Honey

**Published:** 2015

**Authors:** Ali Mohammad Ranjbar, Omid Sadeghpour, Mahnaz Khanavi, Mohammad Reza Shams Ardekani, Hamid Moloudian, Mannan Hajimahmoodi

**Affiliations:** a *Department of Traditional Pharmacy, Faculty of Traditional Medicine, Tehran University of Medical Sciences, Tehran, Iran. *; b *Department of Pharmacognosy, Faculty of Pharmacy, Shahid Sadoughi University of Medical Sciences, Yazd, Iran.*; c *Department of Pharmacognosy, Faculty of Pharmacy, Tehran University of Medical Sciences, Tehran, Iran. *; d *Persian Medicine and Pharmacy Research Center, Tehran University of Medical Sciences, Tehran, Iran. *; e *Department of Drug and Food Control, Faculty of Pharmacy, Tehran University of Medical Sciences, Tehran, Iran.*; f *Food and Drug Administration, Tehran University of Medical Sciences, Tehran, Iran.*

**Keywords:** Honey, IHC, Process, Deslagging, Traditional medicine

## Abstract

Some physicochemical parameters of honey have been introduced by the International Honey Commission to evaluate its quality and origin but processes such as heating and filtering can affect these parameters. In traditional Iranian medicine, deslagging process involves boiling honey in an equal volume of water and removing the slag formed during process. The aim of this study was to determine the effects of deslagging process on parameters of color intensity, diastase evaluation, electrical conductivity, pH, free acidity, refractive index, hydroxy methyl furfural (HMF), proline and water contents according to the International Honey Committee (IHC) standards. The results showed that deslagged honey was significantly different from control honey in terms of color intensity, pH, diastase number, HMF and proline content. It can be concluded that the new standards are needed to regulate deslagged honey.

## Introduction

Honey is a functional food which has been used widely from the past to the present day (1). It consists of glucose, fructose, maltose, sucrose, water, glucuronic acid, lactone, minerals and vitamins (2-3). Honey also contains poly phenolic compounds (4), amino acids, proteins and other components that contribute to its nutritional and medicinal properties (5). Some international organizations such as Codex Alimentarius (6), European Union (7) and International Honey Commission (IHC) (8) have set standards to control this important natural food. There are some papers reporting physicochemical properties of honey and the effects of these parameters on the quality of honey from different origins (9-17). Honey may be treated by heating that the main purposes of the heat-treating are to reduce crystallization and prevent contamination (18-23). There are researches conducted to determine the effects of intensity and duration of the heating process on physicochemical parameters of honey and its medicinal effect (13, 21- 24).

According to the traditional Iranian medicine, honey is consumed as a natural food and medicinal ingredient to prepare unique and complex traditional formulas. It should be noted that deslagged form of honey is used in the traditional formulas. To prepare deslagged honey, it is dissolved in an equal volume of boiling water, boil slowly and then the slag should be removed from the surface. The deslagged honey is suitable form of honey for using in traditional formulas to prevent physicochemical and microbial decompositions. Also deslagging leads to the new consumption due to the change of honey’s temperament such as safe usage in pregnant women and having no side effect in patient with cough. This method was introduced by Galen and applied by medieval scientists such as Avicenna and Razes (25-26).

This study was designed to determine effects of the deslagging process on some important physicochemical parameters of honey.

## Experimental

*Materials and methods*

Five different honey boxes were purchased from the market where mostly sell first material used in traditional pharmacy. The samples were originated from* Anethumgraveolens* L. (Dill),* Astragalus spp*. (milk vetch), *Citrus spp*. (citrus) and the two remain ones were multi floral in origin. All reagents were analytical grade and were purchased from Merck (Darmstradt, Germany).

*Deslagging procedure*

Each honey samples were divided in three sets. The first set was stored at room temperature as control groups. The second set was treated according to the following method described in the literature on traditional Iranian medicine; 500 mL water was added to 500 g of honey. It was warmed slowly to boiling point on a heater and the surface slag was collected by a spatula. Honey was weighed until all water evaporated and then it was cooled at room temperature. The third set was processed in the same way as the second part except that heating was done in a boiling water bath.

*IHC tests*

*Proline*

Proline content is determined calorimetrically after complex formation with ninhydrinas proposed by the harmonized method of International Honey Commission (IHC) (8, 27). 1 mL of 3% (w/v) ninhydrin solution inethylene glycol monomethylether was added to the mixture of formic acid (1 mL) and 0.5 mL of 5% honey solution. 0.5 mL double distilled water and 0.5 mL proline standard solution (0.8 mg/25 mL) were used as the blank and reference respectively. Final mixtures were placed in boiling water for 15 min and then in water bath at 70 ^°^C for 10 min. Finally absorbance was measured 45 min after removal from the bath at 510 nm and an addition of 50% mixture of 2-propanol-water (5 mL). Proline content was calculated by following formula:

Proline (mg/Kg) = (E_s_/E_a_) × (E_1_/E_2_) × 80

Where E_s_ is absorbance of the sample solution, E_a_ is the average of the two absorbances. Measurements for the proline standard solution, E_1_ is the mg of proline taken for the stoke solution, E_2_is the weight of honey in grams and 80 is the dilution factor.

*Diastase*

Diastase activity was measured according to the Schade method presented in IHC with some modification (8). Briefly, a honey solution containing 10 g honey, 5 mL acetate buffer (pH 5.3 and 2.12 M) and 3 mL of NaCl (2.9%) was volumed to 50 mL with double distilled water and incubated at 40 ^◦^C for 15 min. Then 5 mL of starch solution 4% (w/v) at 40 ^◦^C was added to the honey solution and the mixture was returned to the water bath. 0.5 mL of honey-starch mixture was diluted with the appropriate volume of water after 5 min. In the next step, 5 mL diluted iodine solution (0.088 mg/L) was added to the diluted mixture and absorbance was measured at 660 nm. Absorbance measurement continued until 3-4 measurements were taken between 0.465 and 0.155. According to the linear regression equation, the time related to the absorbance equals to 0.235 was calculated and finally diastase number was calculated as follows (28, 29);

DN = 300/t_A=0.235_

Where t_A=0.235_ is the time (28) that predicted absorbance according to the linear regression is 0.235.

*Hydroxy Methyl Furfural (HMF) content*

According to IHC methods, HMF is measurable in 3 ways. The HMF content measurement is based on the absorbance at 284 nm. According to White method a 5 g honey sample is dissolved in approximately 25 mL of water and then 0.5 mL of 15% w/v potassium hexacyanoferrate (30) and 0.5 mL of 30% w/v zinc acetate was added to the medium. Finally it was made up to a volume of50 mL with double distilled water. The mixture was filtered through 0.45 µm filter and first 10 mL of filtrate was discarded. 5 mL of filtrate plus 5 mL of water was pipetted in to a glass tube and after incubation for 1 hour at room temperature, the level of absorbance was determined at 284 and 336 nm. To prepare the blank, 5 mL of 0.2% sodium bisulfite was added to the 5 mL filtrate. If the level of absorbance was greater than 0.6 at 284 nm, the sample and the blank were diluted with water and sodium bisulfite respectively. HMF content (mg/Kg of honey) is calculated as follows;

HMF = (A_284 nm_ - A_336 nm_) × 149.7 × 5 × D. 

Where A_284 nm _and A_336 nm _are absorbance levels of sample solution at 284 and 336 nm respectively, the constant of 149.7 was derived from the molecular weight of HMF and the molar absorptivity of HMF at λ=284 nm and finally D is the dilution factor that is the final volume of the sample solution in mL/10.

*Electrical conductivity (EC)*

EC is calculated as the electric flow of a 20% honey solution (w_dry matter_/v) in terms of mili-Siemens per cm (mS/cm) measured by a conductometer. The temperature was set at 20 ^°^C in a water bath with a thermostat.

*Refractive Index (RI) and water content*

The honey sample was homogenized, transferred to an air-tight flask and then placed in a water bath with a thermostat at 50 ^°^C until all sugar crystals had dissolved. RI was measured by an Abbe refractometer at 20 ^°^C. Water content was obtained from the table cited in the IHC method (8).

*Color intensity*

According to the method developed by Beretta *et al. *(31), absorbance of the honey sample in warm (45-50 ^°^C) distilled water (50% w/v)was measured after filtration through a 0.45 µm filter at two wave length (450 and 720 nm) and the difference between absorbencies was reported as mAU (31).

*PH and freeacidity*

Free acidity was determined by titration to pH 8.3 as follow; A 10 g honey sample was diluted in 75 mL carbon dioxide-free water. The pH was measured by Digital pH-meter and then the solution was titrated by 0.1 M NaOH to pH 8.3. Free acidity in mEq or mmol acid/Kg honey was calculated as follows;

mL of 0.1 M NaOH× 10

## Results

Honey samples were selected from five different origins and processed by deslagging. Then some substantial parameters like color intensity, diastase number, electrical conductivity, pH, free acidity, refractive index, HMF, proline and water content were determined (Table 1). The deslagging process made honey samples more chromatic and eliminated the turbidity. SPSS analysis of the physicochemical parameters showed significant difference between control and processed honey in terms of color intensity, pH, diastase number, HMF and proline content. Color intensity, HMF content and pH increased in processed honey samples but proline content and diastase number were decreased. Diastase number and refractive index were the two parameters that varied significantly between the two tested methods of deslagging. In the case of refractive index, although there was difference between the two methods of processing, group 1 honeys were not different from controlones (α ≤ 0.05). The results are presented in Table 1.

**Table 1:** Physicochemical parameters of control, processed by heater and processed by water bath.

	**Honey**	**Color intensity (mAU)** **± RSD (%)**	**Diastase number (min** ^-1^ **)** **± RSD (%)**	**Electrical conductivity (mS/cm)** **± RSD (%)**	**HMF (mg/Kg)** **± RSD (%)**	**pH** **± RSD (%)**	** Free acidity (mEq/Kg)** **± RSD (%)**	**Proline (mg/Kg)** **± RSD (%)**	**R.I.** **± RSD (%)**	**Water content (%)** **± RSD (%)**
Control^a^	Citrus	91.60 ± 1.09	6.53 ± 0.31	0.447 ± 0.002	118.58 ± 0.84	3.70 ± 0.27	36.00 ± 0.00	270.38 ± 3.70	1.498 ± 0.027	15.40 ± 1.30
	Milk vetch	39.92 ± 1.23	19.65 ± 0.21	0.206 ± 0.016	38.12 ± 2.14	3.95 ± 0.21	24.00 ± 0.00	401.66 ± 2.03	1.497 ± 0.011	15.77 ± 0.30
	Dill	343.86 ± 0.70	8.80 ± 0.42	0.420 ± 0.008	377.64 ± 1.07	3.89 ± 0.21	29.10 ± 0.28	554.62 ± 1.45	1.488 ± 0.011	19.50 ± 0.42
	Multi floral 1	314.17 ± 0.52	5.69 ± 0.57	0.259 ± 0.031	521.95 ± 0.94	3.95 ± 0.21	26.00 ± 0.00	428.44 ± 1.91	1.498 ± 0.017	15.53 ± 0.61
	Multi floral 2	106.57 ± 0.77	5.16 ± 0.79	0.291 ± 0.028	180.12 ± 2.04	4.21 ± 0.19	24.30 ± 0.34	241.05 ± 2.03	1.498 ± 0.019	15.50 ± 0.53
1^b^	Citrus	314.33 ± 0.64	ND^d^	0.447 ± 0.002	1678.53 ± 0.60	3.98 ± 0.25	35.00 ± 0.00	131.79 ± 1.52	1.493 ± 0.020	17.37 ± 0.88
	Milk vetch	185.59 ± 0.44	ND	0.271 ± 0.002	458.23 ± 0.89	5.35 ± 0.15	23.40 ± 0.35	283.96 ± 2.30	1.488 ± 0.003	19.37 ± 0.24
	Dill	793.22 ± 0.31	ND	0.422 ± 0.006	782.60 ± 0.73	4.23 ± 0.19	30.00 ± 0.00	296.05 ± 1.52	1.498 ± 0.020	15.50 ± 0.53
	Multi floral 1	587.96 ± 0.42	ND	0.260 ± 0.031	1687.36 ± 0.48	4.74 ± 0.17	26.00 ± 0.00	204.42 ± 1.60	1.499 ± 0.003	15.07 ± 0.31
	Multi floral 2	387.91 ± 0.42	ND	0.291 ± 0.028	546.12 ± 0.82	4.73 ± 0.17	23.60 ± 0.35	126.09 ± 1.94	1.497 ± 0.000	15.80 ± 0.00
2^c^	Citrus	231.39 ± 0.35	2.95 ± 1.11	0.447 ± 0.002	1920.46 ± 0.43	4.13 ± 0.20	35.50 ± 0.23	145.54 ± 2.82	1.485 ± 0.022	20.43 ± 0.61
	Milk vetch	107.73 ± 0.76	7.31 ± 0.22	0.335 ± 0.007	1039.24 ± 0.79	5.41 ± 0.15	24.40 ± 0.33	295.16 ± 1.38	1.485 ± 0.003	20.60 ± 0.00
	Dill	668.32 ± 0.37	4.18 ± 0.59	0.424 ± 0.019	1389.29 ± 0.59	4.50 ± 0.18	28.30 ± 0.29	351.16 ± 2.33	1.494 ± 0.023	17.03 ± 0.73
	Multi floral 1	527.42 ± 0.46	2.84 ± 0.86	0.266 ± 0.031	1831.74 ± 0.45	4.91 ± 0.17	26.00 ± 0.00	265.87 ± 1.54	1.493 ± 0.014	17.40 ± 0.47
	Multi floral 2	275.21 ± 0.59	2.79 ± 0.88	0.299 ± 0.027	702.24 ± 0.81	4.82 ± 0.17	23.00 ± 0.00	169.83 ± 1.92	1.481 ± 0.006	22.27 ± 0.21

a no processed honey

b deslagged on heater

c deslagged in water bath

d not defined

## Discussion

IHC harmonized tests were done on honey samples from five different origins arranged in three sample sets; control, deslagged by heater (group 1) and deslagged in a boiling water bath (group 2). These tests were selected as international standard test to control honey quality and also to be practical in different laboratories. As shown in Table 1, some parameters in control honey samples did not meet standard levels according to the indicator of honey quality in IHC (8); such as diastase number and HMF content in citrus, multi floral A and B honeys and HMF content in dill honey. Moreover during the deslagging process some quality indicators reduced in all samples that indicate honeys as non-standard. Proline is a kind of amino acid in honey that is produced by bees and is a good indicator for honey quality (8). According to the IHC, proline content should be more than 200 mg/Kg, which was accepted in all the control honey samples in this study. Although it is partly resistant to heating, it was reduced through the deslagging process that can be due to the heating in a complex medium and one of the honey samples with borderline proline content in its control form was ruled following the deslagging process. Therefore the new standard limit for proline content should be introduced for deslagged honey. Diastase is an enzyme that breaks down starch to maltose and its content in the term of min^-1^should be more than 8 (8). In this study three control honey samples were confirmed with diastase numbers but whole samples were rejected during the deslagging process. According to the results, diastase number was significantly different between honey groups 1 and 2.It was completely deactivated during direct heating applied in group 1but reduced its activity to around half the time in group 2. Therefore it can be used in deslagging method specifications. Hydroxymethyl furfural (HMF) is a product of fructose decomposition that can be used as an indicator of shelf life, heating effect and sugar adulteration in honey. According to IHC standards, t should not be more than 40 and 80 mg/Kg in cold and tropical climates respectively. Only one of the control honey samples in this study was considered acceptable according to this criterion but the deslagging process led to a rejection of this sample too. This could be explained by sugar adulteration or unsuitable storage conditions. Comparison between diastase and HMF in group 1 and 2 show that diastase was more affected by direct heating in group 1 while HMF rise was observed in group 2 more than group 1. It can be explained by the time of two processes as HMF content is more sensitive to the time of heating. Deslagging process time was not the same in different kind of honeys but the time consumption in method 2 was near two time in method 1.Electrical conductivity is an indicator employed to determine type among unifloral honey samples (9,14,32,33). It was not changed significantly in both processing showing that it can be used as an indicator for processed unifloral honey. Refractive index and water content are correlated to each other and can be employed to control the residual amount of water in deslagged honey.

Tosi and coworkers have investigated the effect of isothermal and gradient heating processes on physicochemical parameters of honey such as diastase activity and HMF content (21-23). The aim of these studies was to specify a thermal processing condition with the minimal effect on physicochemical honey factors. The effect of both transient and isothermal steps of heating process on honey HMF content in the range of 100 °C to 160 °C were studied in 2002 and results showed that although HMF content has raised during heating process, the initial amount of HMF in control honey did not have any effect on HMF production during the process and that its formation follows the first order kinetics (21). The most severe heating condition with the minimum accepts a cecriteria for diastase number was introduced in 2004 by Tosi and coworkers (23). It was 140 °C during 15 s for transient step and 30 s in isothermal stage. In 2008, diastase activity during heating between 60 °C to 100 °C up to 1200 s was investigated (22). Diastase deactivation was observed in all samples at 100 °C that is similar to present study but it was interesting that diastase was reactivated at medium temperatures during long term heating process (600 and 1200 S). Also the effect of gamma radiation (18, 34, 35), pasteurization, heating by warm air flow, electrical resistance, microwave and heating in condition of reduced air pressure (29,36) on some honey parameters have been investigated. 

## Conclusion

This study aimed to illustrate the effect of deslagging process according to the two specific methods on honey parameters. The results showed color intensity, pH, diastase number, HMF and proline content significantly changed during the processing and therefore deslagged honeys would be rejected according to the current standards. As a result it can be concluded that physicochemical parameters can be used to control the deslagging process but definition of a new standard limits for deslagged honey is needed as deslagged honey is different from virgin honey. It should be noted Tosi and coworkers studies aimed to find optimum conditions with acceptance criteria for mentioned processes that can be suggested to do the same studies for deslagging process.
